# Improved nutrition cues switch from efficiency to luxury phenotypes for a long‐lived ungulate

**DOI:** 10.1002/ece3.2457

**Published:** 2016-09-22

**Authors:** Eric S. Michel, Emily B. Flinn, Stephen Demarais, Bronson K. Strickland, Guiming Wang, Chad M. Dacus

**Affiliations:** ^1^ Department of Wildlife, Fisheries and Aquaculture Forest and Wildlife Research Center Deer Ecology and Management Laboratory Mississippi State University Mississippi State MS USA; ^2^ Department of Wildlife, Fisheries and Aquaculture Mississippi State University Mississippi State MS USA; ^3^ Mississippi Department of Wildlife Fisheries and Parks Jackson MS USA

**Keywords:** canalization, efficiency phenotype, luxury phenotype, maternal effects, phenotypic plasticity, phenotypic variation, white‐tailed deer

## Abstract

Cervid phenotype can be categorized as efficiency, which promotes survival but not extravagant growth, or luxury which promotes growth of large weaponry and body size. Although nutritional variation greatly influences these phenotypic forms, the potential for subspecies‐linked genetic or founder effects from restocking efforts of harvested species has not been eliminated. We measured intergenerational phenotypic change of males in response to improved nutrition in three captive‐reared populations of white‐tailed deer. Study animals were offspring of females captured from three regions displaying variation in antler and body size as well as nutritional variation. We fed all animals a high‐quality diet and measured antler and body size for two generations. We predicted that improved long‐term nutrition would cue a switch from efficiency to luxury phenotype for all populations and that regional compensation of antler and body size would occur. Improved nutrition positively influenced all measures of antler and body size; however, changes varied in magnitude. Antler size was more responsive than body size. Improved nutrition also facilitated regional compensation of antler size and partial compensation of body size. Our results show that improved long‐term nutrition cues a shift from efficiency to luxury phenotype in a long‐lived cervid with weaponry being more responsive than body size. Compensation of antler size suggests that weaponry is greatly influenced by nutrition and is not restricted by subspecies‐linked genetic or founder effects from restocking efforts related to our regional populations. Therefore, strategies to improve cervid antler and body size should include habitat management that elevates long‐term diet quality.

## 1 Introduction

Environmental cues during gestation and lactation influence a wide variety of offspring phenotypic characteristics independent of an offspring's genotype (Bernardo, 1996; Forchhammer, Clutton‐Brock, Lindstrom, & Albon, 2001; Freeman, Larsen, Clegg, & McMillan, 2013; Mech, Nelson, & McRoberts, 1991). Such environmental cues can also impact offspring stress levels, reproduction, immune system function (Bian et al., 2015; Triggs & Knell, 2012), behavior, and subsequent survival (Kerr, Boutin, LaMontagne, McAdam, & Humphries, 2007; Skibiel, Dobson, & Murie, 2009; Storm & Lima, 2010). Thus, spatial and temporal environmental variation may indirectly and directly influence individual fitness as well as population dynamics (Kruuk et al., 2000; McAdam, Boutin, Réale, & Berteaux, 2002; Räsänen & Kruuk, 2007).

**Figure 1:**
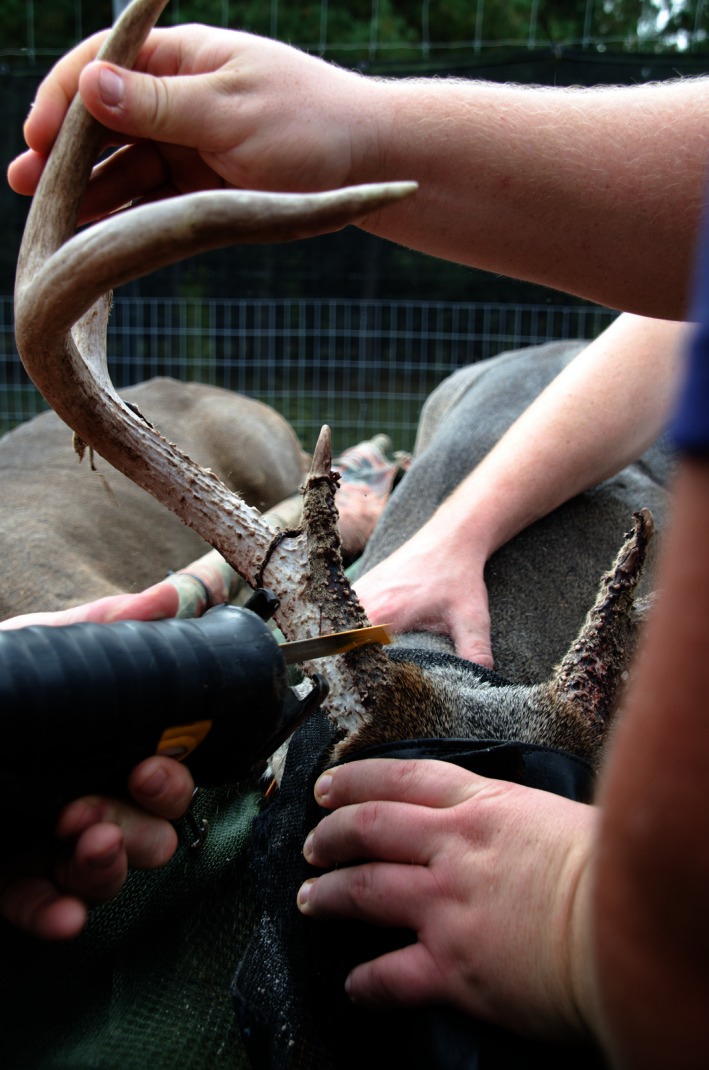
Removal of antlers from male white‐tailed deer for data collection

Environmental cues that influence intergenerational phenotype may alter the evolutionary trajectory of a population (McAdam et al., 2002; Räsänen & Kruuk, 2007). For instance, when nutritional quality limits populations, individuals display an efficiency phenotype that promotes survival, but not the production of extravagant weaponry such as large antlers, horns, and/or body sizes (Geist, 1989). Conversely, larger weaponry and body sizes should be prevalent when nutritional resources are abundant (i.e., a luxury phenotype will be displayed; Geist, 1989). Efficiency and luxury phenotypes may further dichotomize because weaponry and body size influence reproduction (Clutton‐Brock, Guinness, & Albon, 1982; Coltman, Festa‐Bianchet, Jorgenson, & Strobeck, 2002; Festa‐Bianchet, 2012; Kie et al., 2013; Lidgard, Bowen, & Boness, 2012). Therefore, individuals displaying optimum phenotypes should have improved fitness (Fig. 1).

Populations of the same species display efficiency and luxury phenotypes which may have complicated historical morphometric‐based taxonomy. For example, Strickland and Demarais (2000) reported a wide range in antler and body sizes of adult male white‐tailed deer (*Odocoileus virginianus*) across Mississippi, USA, with some populations about one‐third larger than others. This phenotypic variation is seemingly related to variation in forage quality reported by Jones, Demarais, Strickland, and Edwards (2008) with the largest males found in areas with the greatest quantity and quality of forages. However, genetic bottlenecks, founder effects, or genetic remnants from white‐tailed deer restoration (DeYoung et al., 2003; Sumners et al., 2015) could be partially responsible for some of the observed phenotypic variation. Some findings of Strickland and Demarais (2000) support the subspecies classification of white‐tailed deer in southeastern Mississippi (*O. v. osceola*; Baker, 1984), which was based on phenotypic size with *O. v. osceola* described as being smaller than their *O. v. virginianus* counterparts (Barbour & Allen, 1922). However, this subspecies classification may be unnecessary if phenotypic variation among populations is simply related to diet quality rather than genetic differences. Increases in phenotypic characteristics as a response to improved nutrition have been reported for some ungulates (red deer; *Cervus elaphus,* roe deer; *Capreolus capreolus*; reviewed in Geist, 1986; white‐tailed deer*,* Monteith, Schmitz, Jenks, Delger, & Bowyer, 2009); however, the relative influence of population‐level genetics on white‐tailed deer phenotype is still uncertain.

Our goal was to measure phenotypic change as a response to improved nutrition during two generations using captive male white‐tailed deer. Wild populations of white‐tailed deer are generally overpopulated across their range which can lead to nutritional degradation of their habitat (reviewed in Côté, 2011; Demarais, Miller, & Jacobson, 2001). We therefore hypothesized that a high‐quality diet (i.e., 20% crude protein deer pellets fed *ad libitum*) would increase size of phenotypic characteristics by alleviating potential lagging maternal effects due to nutritional restrictions experienced in the wild (Geist, 1986; Monteith et al., 2009). We also hypothesized that regional compensation of phenotypic characteristics would occur in the second generation (Monteith et al., 2009). We further hypothesized that not all phenotypic characteristics would respond to improved nutrition at the same rate or magnitude. For example, skeletal structures seemingly display a high level of canalization (Benowitz‐Fredericks, Kitaysky, & Thompson, 2006; Simard, Côté, Weladji, & Huot, 2008; Waddington, 1957) and may not respond to improved nutrition. However, weaponry and body mass are known to influence reproduction positively (Bartoš & Bahbouh, 2006; Clutton‐Brock et al., 1982; Coltman et al., 2002) and, such, should display larger increases than skeletal structures when high‐quality nutrition is abundant. Therefore, we predicted antler size and body mass would display a larger increase compared to skeletal structures. Alternatively, phenotype could be limited by population‐level genetics that limit the response to improved nutrition within one generation. Understanding the relative effects of population‐level genetics and/or nutrition on white‐tailed deer phenotypic expression will also greatly help refine management strategies aimed at increasing phenotypic size for this species.

## 2 Materials and Methods

### 2.1 Source populations

**Figure 2:**
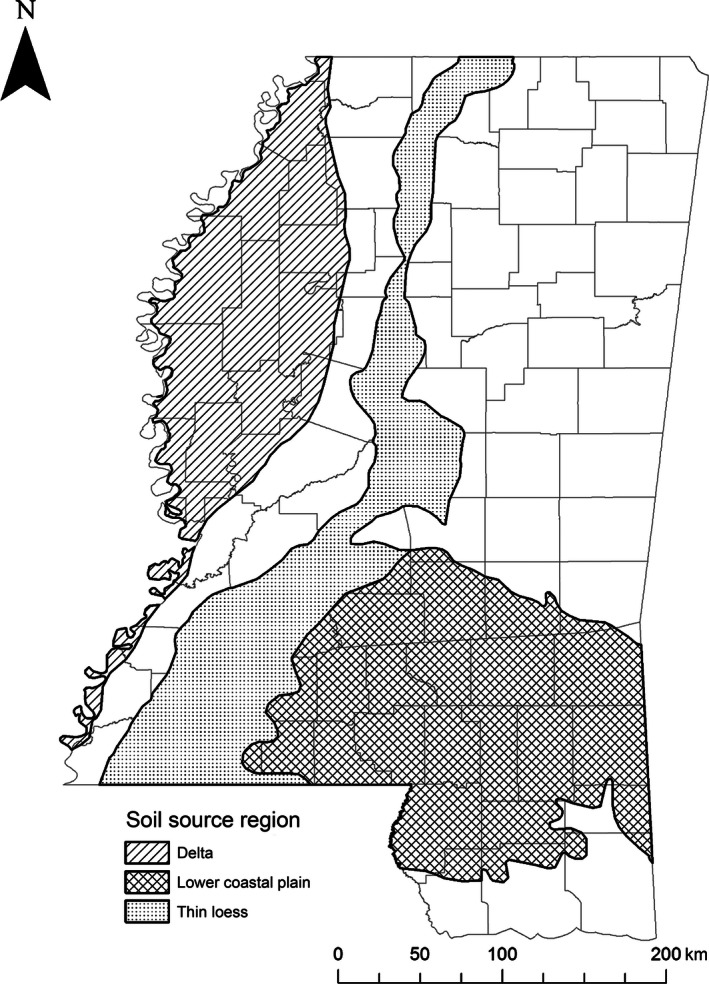
Physiographic regions of Mississippi where pregnant dams and fawns were captured

To incorporate the range of inherent genetic and habitat variation, we captured deer from 29 sites located on public wildlife management areas and private lands that were part of the Deer Management Assistance Program (Guynn, Mott, Cotton, & Jacobson, 1983) throughout three soil source regions in Mississippi, USA (Fig. 2). The Delta soil region comprises nearly 14% of total land area of Mississippi, USA, and is classified as a high‐quality soil region with agriculture as the primary land use (e.g., cotton, soybean, corn, rice; Pettry, 1977; Snipes et al., 2005). The mean soil productivity value for capture sites in the Delta region was 10.1 (range 0–19; Soil Drainage and Productivity Index Map, http://foresthealth.fs.usda.gov/soils/PIMap). The Delta soil region and all study animal source populations were within the distribution of *O. v. virginianus* (Baker, 1984). The Thin Loess soil region (upper and lower Thin Loess combined) comprises almost 14% of total land area of Mississippi, USA, and is considered a medium‐quality soil region. Its primary land use is also agriculture, although not as prevalent as in the Delta (Pettry, 1977; Snipes et al., 2005). The mean soil productivity value for capture sites in the Thin Loess region was 8.8 (range 0–19; Soil Drainage and Productivity Index Map, http://foresthealth.fs.usda.gov/soils/PIMap). The Thin Loess region and all study animal source populations were within the distribution of *O. v. virginianus* (Baker, 1984). Lastly, the Lower Coastal Plain (LCP) soil region comprises nearly 22% of Mississippi. This area is classified as a low‐quality soil region and has leaching issues, limiting most land uses to pine (*Pinus* spp.) production and livestock grazing (Pettry, 1977; Snipes et al., 2005). The mean soil productivity value for capture sites in the LCP region was 3.7 (range 0–19; Soil Drainage and Productivity Index Map, http://foresthealth.fs.usda.gov/soils/PIMap). The LCP soil region overlaps the geographical distribution of *O. v. osceola,* and four of the six source populations were in or within 21 km of this distribution (Baker, 1984). This subspecies was described as being smaller than *O. v. virginianus* (Barbour & Allen, 1922).

### 2.2 Study area

We brought all wild‐caught animals to the Mississippi State University Rusty Dawkins Memorial Deer Unit (MSU Deer Unit). The MSU Deer Unit is located in Oktibbeha County, Mississippi, USA, and is subdivided into five 0.4‐ to 0.8‐ha pens. We housed ≥5.5‐month‐old males at satellite facilities located near Macon, Noxubee County; Kosciusko, Attala County; Utica, Copiah County; and Morton, Scott County, Mississippi, USA. Each satellite facility consisted of two 0.7‐ha pens. We raised all deer on a high‐quality diet comprised of 20% crude protein deer pellets (Purina AntlerMax Professional High Energy Breeder 59UB, Purina, MO, USA) fed ad libitum. Available forages within pens included Durana Clover and Max‐Q Fescue (Pennington Seed Co., Georgia, USA) along with volunteer grasses and forbs. All facilities had similar husbandry practices.

### 2.3 First and second generations

We produced two generations of offspring by allowing first‐generation males to naturally breed first‐generation females from the same soil source region (e.g., Delta males bred Delta females, Thin Loess males bred Thin Loess females, and LCP males bred LCP females). Each year, we placed two males with 7–16 females, for an average breeding sex ratio of one male per eight females. Females produced offspring for multiple years but typically with different sires each year. We categorized two groups of deer as first‐generation (F1) individuals. We considered deer caught as 5.5‐month‐old fawns from the wild as well as offspring born in the MSU Deer Unit from wild‐born mothers as F1 individuals. All F1 deer were raised on the same high‐quality diet ad libitum the remainder of the project. Second‐generation (F2) deer were offspring of F1 deer, were raised in captivity from birth, and had access to the same high‐quality diet ad libitum as their F1 parents.

### 2.4 Data collection

#### 2.4.1 Newborn fawns

We searched the MSU Deer Unit daily for fawns starting on 1 June from 2005 to 2010. We uniquely marked fawns within 1 day of birth with medium plastic ear tags (Allflex, Texas, USA), measured body mass (nearest 0.01 kg) using a digital vertical hanging scale (Pelouze, Illinois, USA), measured total body length and hind food length to the nearest mm, and collected hair samples or ear notches for DNA analysis. DNA Solutions (Oklahoma, USA) assigned parentage of fawns using DNA based on a proprietary, nonstatistical custom structured query language database known as the DNA Solutions Animal Solutions Manager (DASM^©^). In the pairwise allele comparison, DNA Solutions assigned parentage when they excluded all but one sire and one dam based upon a shared allele from each parent at all loci tested (B. G. Cassidy, DNA Solutions, personal communication). We administered 2 cc's of Clostridium Perfringens types C and D Toxoid Essential 3 and Clostridium Perfringens types C and D Antitoxin Equine Origin (Colorado Serum Company, Colorado, USA) subcutaneously and 0.3 cc/kg of ivermectin in propylene glycol (Mississippi State University Veterinarian School, Mississippi, USA) orally to each fawn.

#### 2.4.2 Juveniles

We chemically immobilized juveniles approximately 5.5 months after their average region‐specific birth date. We used a 2:1 mixture of Telazol (Fort Dodge Animal Health, Iowa, USA) and xylazine HCl (Phoenix Scientific, Missouri, USA) with an approximate dosage of 6.6 mg/kg via cartridge‐fired dart (Pneu‐Dart Inc, Pennsylvania, USA). We recorded the same measurements for juveniles that we collected from newborn fawns, marked juveniles with a large plastic tag in each ear (Allflex), and administered size‐appropriate amounts of the antibiotic Nuflor ^™^ (Schuering‐Plough Animal Health Corp., New Jersey, USA), the endectocide ivermectin (Norbrook Laboratories, LTD., Down, Northern Ireland, UK), the clostidrial vaccine Vision 7 with SPUR (Ivesco LLC, Iowa, USA), and the leptospirosis vaccine Leptoferm‐5 (Pfizer, Inc., New York, USA) to individuals from all regions. We reversed the effects of xylazine HCl with 0.125 mg/kg yohimbine HCl (Kreeger, 1996) or 4.0 mg/kg tolazoline HCl (Miller et al., 2004). We then transported the juvenile males to one of four satellite facilities. Each satellite facility received an equal, random sample of juveniles from each soil source region.

#### 2.4.3 Adults

We chemically immobilized adult males (≥1 year‐old) for data collection during October and November, 2005–2010. We repeated the same prophylactics and morphometric measurements collected from neonates and juveniles. We also measured antler size of adult males by measuring the inside spread, basal circumference, and beam length of antlers prior to their removal. We removed antlers approximately 3 cm above the burr with a reciprocating saw or diamond wire but did not remove antlers less than 3 cm long (Fig. 1). We weighed antlers to the nearest 0.1 g and assigned a minimal critical antler mass of 1 g for first‐year animals with antlers shorter than 3 cm. We calculated an antler score similar to the gross nontypical Boone and Crockett score (Nesbitt, Wright, Buckner, Byers, & Reneau, 2009), but measured less than four circumferences when antlers contained less than three tines. For example, a main beam with two typical points included only three circumference measurements. We also included body mass and antler score from individuals harvested from Mississippi, USA, as reference points. We calculated an estimated live weight of individuals by multiplying the eviscerated body mass reported by Strickland and Demarais (2000) by 1.285. We used the same antler measurements used by Strickland and Demarais (2000) to calculate an antler score index to derive mean, 3.5‐year‐old antler scores for each regional population by applying those measurements to a predictive equation (Strickland et al., 2013). The Mississippi State University Institutional Animal Care and Use Committee approved all capture, handling, and marking techniques under protocols 04–068, 07–036, 10–033, and 13–034.

### 2.5 Data analysis

We used an animal model within the Monte Carlo Markov Chain generalized linear mixed model (MCMCglmm) framework in the MCMCglmm package in Program R (R Development Core Team 2015, version 3.1.3; Hadfield, 2010) to estimate the influence of long‐term, high‐quality nutrition (indexed by generation, a categorical variable) on male white‐tailed deer phenotype. Using an animal model allowed us to account for any variation in phenotype related to the sire and dam. We included animal ID as a random effect, which accounted for multiple measurements of each individual. Body mass and antler size varie by the soil region where we obtained our source populations (Strickland & Demarais, 2000), so we included soil source region as a fixed effect. Body mass and antler size are known to increase with age so we also included age as a fixed effect. Examining the interaction between generation and age would have been informative, but sample size varied for each generation, region, and age class and was inadequate to assess this interaction (Table S1). We considered variables to be significant if the 95% credible interval (95% CI) excluded 0 (Lesaffre & Lawson, 2012). For each model, we ran two chains with uninformative priors and 100,000 iterations for each chain. We sampled every 10th iteration after a 50,000‐iteration burn‐in period. We examined trace plots for convergence of each variable as well as for convergence between chains. We confirmed there was no autocorrelations between iterations within each model. We then used the model parameters to predict means for each phenotypic characteristic using the MCMCglmm.predict function in Program R.

## 3 Results

**Figure 3:**
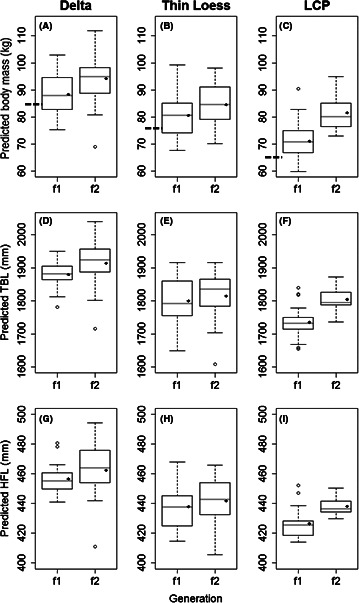
Generational improvement of median body mass, total body length (TBL), and hind foot length (HFL) for captive 3.5‐year‐old male white‐tailed deer housed in Noxubee, Attala, Copiah, and Scott County, Mississippi, USA. Dashed line on the *y*‐axis represents mean body mass of harvest data collected from Mississippi, USA, and is used for comparison to first and second generations. The black diamond represents the predicted mean. Whiskers indicate minimum and maximum values, while open circles indicate outliers

**Figure 4:**
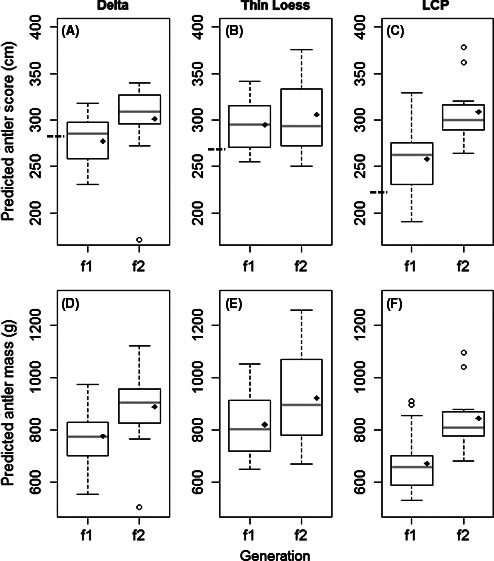
Generational improvement of median antler characteristics for captive 3.5‐year‐old male white‐tailed deer housed in Noxubee, Attala, Copiah, and Scott County, Mississippi, USA. Dashed line on the *y*‐axis represents mean antler score of harvest data collected from Mississippi, USA, and is used for comparison to first and second generations. The black diamond represents the predicted mean. Whiskers indicate minimum and maximum values, while open circles indicate outliers

All phenotypic characteristics increased in size from first to second generation. Quality long‐term nutrition positively influenced phenotype (95% CI above 0; Table 1; Figs 3 and 4). Age also positively affected phenotype (95% CI above 0; Table 1).

**Table 1:** MCMCglmm models describing the influence of generation (F2), age, and region (regionLoess, regionLCP) on phenotypic characteristics. We coded generation and region as categorical variables and age as a continuous variable. The intercept represents first‐generation (F1), 1‐year‐old Delta males and is considered a reference term for comparison of generation, age, and regional soil source population

Response variable										
	Body mass		Hind foot length		Total body length		Antler score		Antler mass	
	Beta	95% CI	Beta	95% CI	Beta	95% CI	Beta	95% CI	Beta	95% CI
Intercept	41.77	37.71 to 46.02	428.05	421.24 to 434.94	1602.46	1568.88 to 1637.57	−6.78	−25.73 to 14.57	−267.74	−347.25 to −186.81
F2	6.39	2.79 to 10.08	8.11	1.73 to 14.29	37.45	6.48 to 67.83	24.84	7.29 to 43.78	104.01	34.99 to 177.03
Age	15.41	14.66 to 16.17	8.64	7.41 to 9.79	92.03	85.16 to 98.76	95.28	90.85 to 99.82	351.24	332.14 to 370.81
regionLCP	−14.15	−19.06 to −9.060	−24.42	−32.58 to −14.70	−122.86	−166.64 to −80.12	−9.96	−35.19 to 14.23	−82.98	−173.85 to 7.78
regionLoess	−10.65	−15.92 to −5.76	−22.16	−31.07 to −13.70	−106.76	−151.39 to −67.01	−0.31	−25.86 to 23.20	10.39	−76.6 to 105.93

Although all phenotypic characteristics increased in size after two generations of improved nutrition, there was still regional variation among populations for some characteristics (Table 1; Fig. 3). Soil source region was a significant predictor for body mass (95% CI above 0) and both skeletal measurements (95% CI above 0), indicating that Delta males grew larger bodies than Thin Loess and LCP males. However, soil source region was not a significant predictor for antler size or mass (95% CI included 0), suggesting there was no longer regional variation of weaponry.

We observed variation in the magnitude of increase for phenotypic characteristics. For example, antler mass and score were generally more sensitive to improved nutrition compared to body mass and skeletal size. Antler mass increased 2.5 times more than body mass and about 15 times more than total body length (Table 2) for Thin Loess males. Males from the other regional populations displayed similar increases from first to second generation for each phenotypic characteristic.

**Table 2:** Percent increase in morphometrics from first to second generation of captive 3.5‐year‐old male white‐tailed deer housed in Noxubee, Attala, Copiah, and Scott County, Mississippi, USA

Variable	Delta			Thin loess			LCP		
	F1 Mean	F2 Mean	% Increase	F1 Mean	F2 Mean	% Increase	F1 Mean	F2 Mean	% Increase
3.5 years									
Body Mass (kg)	88.3	94.2	6.7	80.5	84.4	4.8	70.9	81.4	14.8
Total Body Length (mm)	1879.9	1913.9	1.8	1799.9	1814.7	0.8	1735.0	1804.2	4.0
Hind Foot Length (mm)	456.5	462.4	1.3	437.9	441.8	0.9	426.4	438.0	2.7
Antler Score (cm)	277.2	301.1	8.6	294.8	306.0	3.8	258.1	308.9	19.7
Antler Mass (g)	777.0	888.9	14.4	821.5	921.3	12.1	671.9	845.1	25.8

Magnitude of generational improvement also varied among regional populations. Males from the LCP regional population increased most, as they displayed up to a two times larger increase from first to second generation compared to the Delta and up to a five times larger increase compared to the Thin Loess regional populations (Table 2). LCP males displayed a 15–25% increase in body mass, antler score, and antler mass from first to second generation, while Delta and Thin Loess males increased only 7–14% and 5–13%, respectively (Table 2). Total body length and hind foot length followed similar patterns of increase as LCP males displayed about a 3–4% increase from first to second generation while Delta males displayed about a 1–2% increase and Thin Loess males displayed about a 1% increase. We found similar patterns when examining changes in body mass and antler score from harvested to second‐generation individuals. LCP males increased body mass and antler score about 24 and 38%, respectively (Table 3). Thin Loess males displayed about a 10 and 16% increase and Delta males displayed about an 11 and 5% increase for body mass and antler score, respectively.

**Table 3:** Percent increase in body mass and antler size from 3.5‐year‐old male white‐tailed deer harvested in Mississippi, USA, to a second generation of captive 3.5‐year‐old male white‐tailed deer housed in Noxubee, Attala, Copiah, and Scott County, Mississippi, USA, and raised on optimum nutrition

Variable	Delta			Thin loess			LCP		
	Harvest mean	F2 Mean	% Increase	Harvest mean	F2 Mean	% Increase	Harvest mean	F2 Mean	% Increase
Body mass (kg)	85.1	94.2	10.7	76.6	84.4	10.2	65.5	81.4	24.3
Antler score (cm)	287.8	301.1	4.6	263.4	306.0	16.2	224.2	308.9	37.8

## 4 Discussion

Our results support our hypothesis that high‐quality nutrition would positively influence captive male white‐tailed deer phenotype as all regional populations switched from efficiency to luxury forms. This result supports previous studies of several species (*Ursus americanus*,* Ursus arctos*, Welch, Keay, Kendall, & Robbins, 1997; *Liasis fuscus,* Madsen & Shine, 2000; *Larus michahellis*, Saino et al., 2010) where nutritional quality influenced expression of several phenotypic characteristics. Additionally, phenotypic changes we report occurred within an ecological timeframe allowing for new phenotypic optimums to occur as the three populations experienced a novel environment (Ghalambor, McKay, Carroll, & Reznick, 2007; Pigliucci, Murren, & Schlichting, 2006). A population's ability to respond to changes in nutritional quality and quantity is vital as nutrition ultimately affects reproduction and survival (Cook et al., 2004; Lomas & Bender, 2007; Parker, Barboza, & Gillingham, 2009).

Nutritional cues that positively or negatively affect offspring while in utero or during dependency are generally referred to as maternal effects (Bernardo, 1996). Maternal effects can be thought of as a mother “communicating” the environment with her offspring. This “communication” allows for offspring to display a phenotype suitable for the environment they are born into when the environment is predictable (Mousseau & Fox, 1998). By providing high‐quality nutrition ad libitum*,* we simulated a predictable, high‐quality environment allowing offspring to display a phenotype consistent with their maternal environment, a result previously reported for white‐tailed deer by Monteith et al. (2009). This phenotypic plasticity, potentially mediated by maternal effects, likely explains the widespread distribution of whitetails across the New World (discussed in Wolverton, Lyman, Kennedy, & La Point, 2009).

Epigenetic changes, the heritable changes in gene expression and function that cannot be explained by changes in DNA sequence (Bird, 2007; Bossdorf, Richards, & Pigliucci, 2008; Richards, 2006), are a likely mechanism for maternal effects. Simply put, epigenetic variation can be directly influenced by the environment, thus influencing an individual's phenotype and may be inherited by future generations (Bossdorf et al., 2008; Powledge, 2011). For example, in laboratory mice, maternal diet influenced offspring phenotypes such as coat color (Waterland & Jirtle, 2003; Wolff, Kodell, Moore, & Cooney, 1998) and tail straightness (Waterland et al., 2006). Epigenetics are important because they explain some heritable phenotypic variation in natural populations that are not explained by differences in DNA sequence (Bossdorf et al., 2008) and may provide insight into the plasticity of animals (Bossdorf et al., 2008). We hypothesize white‐tailed deer phenotypes are influenced by epigenetic processes.

Our results also suggest that phenotypic‐based subspecies classification may be inappropriate for some mammalian species. The dramatic increase in antler and body size that we report for the LCP regional population suggests that phenotype may not be restricted by genetics; phenotypic differences between *O. v. virginianus* and *O. v. osceola* may instead be related to environmental differences. This further supports the results of DeYoung et al. (2003) who found no genetic differences among white‐tailed deer subspecies in Mississippi. Therefore, caution should be used when assigning subspecies classifications for mammals (Geist, 1989).

We found partial support of our hypothesis that we would observe regional compensation for all phenotypic characteristics. Body mass and skeletal measurements were greatest for Delta males but did not differ between Thin Loess and LCP males. Our results are consistent with those of Monteith et al. (2009) who found that body mass still varied after two generations of improved nutrition between two populations of white‐tailed deer originating from South Dakota, USA. There are three possible explanations for this result. First, these differences may indicate possible genetic differences among populations that cannot be overcome by improving nutrition. Second, more than two generations of improved nutrition may be needed for full regional compensation to occur. Geist (1986) suggested that four generations of improved nutrition may be needed for white‐tailed deer to display their full genetic potential. Lastly, compensation of body mass may occur for Thin Loess and LCP males as asymptotic body mass is not reached until 4.5 years of age for male white‐tailed deer (Strickland & Demarais, 2000); therefore, these individuals may display increased growth rates over the next year (similar to *Rupicapra rupicapra*; Rughetti & Festa‐Bianchet, 2010) allowing for full regional compensation to occur. Nevertheless, antler score and antler mass did not vary among regional populations after two generations of improved nutrition. This result supports previous research of ungulates (reviewed in Geist, 1986; Monteith et al., 2009) and suggests antler size variation among harvested populations is due to regional variation in nutritional quantity and quality (Jones et al., 2008, 2010) as well as regional variation in land use (Strickland & Demarais, 2008). Regional variation of body, but not antler size, may also indicate differences in plasticity of white‐tailed deer phenotypic characteristics once quality nutrition is available.

In addition to our results for antler score and body mass being similar to Monteith et al. (2009), we found support for our prediction that magnitude of change would vary among phenotypic characteristics as we identified a clear hierarchy of growth prioritization, a result that Monteith et al. (2009) did not document. Antler mass displayed the largest increases followed by antler score, body mass, and finally skeletal characteristics. Increases in antler mass suggest that individuals allocate nutritional resources toward increasing antler strength over antler size. Increased antler mass reduces the probability of breakage (Landete‐Castillejos et al., 2010) and could therefore increase access to mates compared to antler size alone as a visual indicator of male quality (reviewed in Demarais & Strickland, 2011). Concomitantly, these differential rates of change suggest skeletal characteristics display a greater level of canalization than weaponry and body mass. For example, skeletal characteristics displayed minimal change (≤3.9%) from first to second generation; however, antler and body mass increased up to about 25 and 15%, respectively. These results support other studies assessing canalization of skeletal structures. Simard et al. (2008) found white‐tailed deer body mass decreased with a decline in nutritional quality, but hind foot length did not change. Benowitz‐Fredericks et al. (2006) also reported that tarsus length was less likely to be influenced by nutritional intake compared to other phenotypic characteristics for the common Murre (*Uria aalge*). Therefore, skeletal structures are likely highly prioritized during growth regardless of the nutritional environment an individual experiences or may simply take additional time to change (Geist, 1989).

Different levels of canalization among phenotypic characteristics are a potential adaptation to increase male reproductive success (Geist, 1989; Kruuk et al., 2002). Weaponry and body mass are known to influence access to mates (Clutton‐Brock et al., 1982; Festa‐Bianchet, 2012). Antlers are cast and regrown on an annual basis (Demarais & Strickland, 2011); thus, increasing antler size within a given year when nutritional quality allows for it may improve access to mates without having to expend resources producing large antlers in subsequent years if resources are limited. However, body mass is less sensitive to environmental changes compared to antler size, and thus more easily reproducible. An increase in weaponry is therefore advantageous when resources are abundant, but not necessarily when resources are limited (Geist, 1989). These adaptations could potentially allow for an individual to increase annual reproductive success without jeopardizing long‐term reproductive success although future studies are needed to assess these relationships.

Regional variation in phenotypic change is not explained by differences in nutritional quality experienced in the wild. The greater phenotypic improvement by the LCP regional population is intuitive, as deer from this region experienced a greater nutritional limitation in the wild compared to Delta and Thin Loess regional populations (Jones et al., 2008). However, phenotypic improvements for the Delta regional population were unexpected. Deer from the Delta regional population display larger phenotypic characteristics in the wild compared to deer from the Thin Loess and LCP regional populations (Strickland & Demarais, 2000). Deer from the Delta regional population also benefit from high‐quality natural forages (Jones et al., 2008, 2010) and land‐use practices that further promote additional high‐quality forage production (agriculture; Strickland & Demarais, 2008). However, the increased body mass and antler size for the Delta regional population indicate that nutritional quality and/or quantity is lacking in the wild. Therefore, caution must be used when assessing nutritional quality as populations may benefit from improved nutrition even when nutrition appears to be adequate.

## 5 Conclusion

Our results show that nutritional improvements cue a switch from efficiency to luxury phenotypes for three populations of a long‐lived cervid. Nutritional improvements also facilitated full compensation of antler size and partial compensation of body size among populations. This switch in phenotype may influence the evolutionary trajectory of a population, as males with the largest antlers and heaviest body masses may breed more than those with smaller antlers and lighter body masses. If so, this would promote increases in antler and body size as these are heritable traits (Kruuk et al., 2002; Réale, Festa‐Bianchet, & Jorgenson, 1999). We report substantial phenotypic increases after two generations of improved nutrition, which suggests that a potential shift in evolutionary trajectory may occur on an ecological time scale for populations that experience stable nutrition. Therefore, managers with goals aimed at increasing antler and body size should focus efforts on improving nutritional quality as white‐tailed deer phenotype is seemingly not restricted by population‐level genetics. Evaluating past and present nutritional environments when assessing phenotypic variation will also guide management decisions as past environments influence current phenotypes.

## Funding Information

Federal Aid in Wildlife Restoration Act administered through the Mississippi Department of Wildlife, Fisheries and Parks (Grant/Award Number: “W‐48‐61”).

## Conflict of Interest

None declared.
