# Supplementary material for: Improved nutrition cues switch from efficiency to luxury phenotypes for a long‐lived ungulate
Source: Ecol Evol. 2016 Sep 22;6(20):7276–85. doi: 10.1002/ece3.2457 (PMC5127705; doi:10.1002/ece3.2457)
Supplement: Supplementary file 1 [file ECE3-6-7276-s001.docx]

Table S1. Sample sizes for captive male white-tailed deer from three regional soil source populations spanning two generations.

|  |  |  |  |  |  |  |  |  |  |
| --- | --- | --- | --- | --- | --- | --- | --- | --- | --- |
|  |  | Delta | |  | Thin Loess | |  | LCP | |
| Variable |  | F1 (n) | F2 (n) |  | F1 (n) | F2 (n) |  | F1 (n) | F2 (n) |
| 1.5 years |  |  |  |  |  |  |  |  |  |
| Body Mass (kg) |  | 22 | 18 |  | 17 | 25 |  | 27 | 18 |
| Antler Score (cm) |  | 22 | 19 |  | 17 | 25 |  | 27 | 18 |
| Antler Mass (g) |  | 22 | 19 |  | 17 | 25 |  | 27 | 18 |
| Hind Foot Length (mm) |  | 22 | 18 |  | 17 | 25 |  | 27 | 18 |
| Total Body Length (mm) |  | 22 | 18 |  | 17 | 25 |  | 27 | 18 |
|  |  |  |  |  |  |  |  |  |  |
| 2.5 years |  |  |  |  |  |  |  |  |  |
| Body Mass (kg) |  | 19 | 16 |  | 15 | 23 |  | 25 | 16 |
| Antler Score (cm) |  | 19 | 16 |  | 15 | 23 |  | 25 | 16 |
| Antler Mass (g) |  | 19 | 16 |  | 15 | 23 |  | 23 | 16 |
| Hind Foot Length (mm) |  | 19 | 16 |  | 15 | 23 |  | 25 | 16 |
| Total Body Length (mm) |  | 19 | 16 |  | 15 | 23 |  | 25 | 16 |
|  |  |  |  |  |  |  |  |  |  |
| 3.5 years |  |  |  |  |  |  |  |  |  |
| Body Mass (kg) |  | 15 | 15 |  | 14 | 19 |  | 21 | 11 |
| Antler Score (cm) |  | 15 | 15 |  | 14 | 21 |  | 22 | 11 |
| Antler Mass (g) |  | 15 | 15 |  | 14 | 21 |  | 22 | 11 |
| Hind Foot Length (mm) |  | 15 | 15 |  | 14 | 21 |  | 22 | 11 |
| Total Body Length (mm) |  | 15 | 15 |  | 14 | 20 |  | 22 | 11 |
